# Aggregation of Nontuberculous Mycobacteria Is Regulated by Carbon-Nitrogen Balance

**DOI:** 10.1128/mBio.01715-19

**Published:** 2019-08-13

**Authors:** William H. DePas, Megan Bergkessel, Dianne K. Newman

**Affiliations:** a Division of Biology and Biological Engineering, California Institute of Technology, Pasadena, California, USA; b Division of Geological and Planetary Sciences, California Institute of Technology, Pasadena, California, USA; Washington University School of Medicine in St. Louis

**Keywords:** *Mycobacterium*, biofilms, carbon metabolism, nitrogen metabolism, physiology

## Abstract

Nontuberculous mycobacteria (NTM) are emerging opportunistic pathogens that colonize household water systems and cause chronic lung infections in susceptible patients. The ability of NTM to form surface-attached biofilms in the nonhost environment and corded aggregates *in vivo* is important to their ability to persist in both contexts. Underlying the development of these multicellular structures is the capacity of mycobacterial cells to adhere to one another. Unlike most other bacteria, NTM spontaneously and constitutively aggregate *in vitro*, hindering our ability to understand the transition between planktonic and aggregated cells. While culturing a model NTM, Mycobacterium smegmatis, in rich medium, we fortuitously discovered that planktonic cells accumulate after ∼3 days of growth. By providing selective pressure for bacteria that disperse earlier, we isolated a strain with two mutations in the oligopeptide permease operon (*opp*). A mutant lacking the *opp* operon (Δ*opp*) disperses earlier than wild type (WT) due to a defect in nutrient uptake. Experiments with WT M. smegmatis revealed that growth as aggregates is favored when carbon is replete, but under conditions of low available carbon relative to available nitrogen, M. smegmatis grows as planktonic cells. By adjusting carbon and nitrogen sources in defined medium, we tuned the cellular C/N ratio such that M. smegmatis grows either as aggregates or as planktonic cells. C/N-mediated aggregation regulation is widespread among NTM with the possible exception of rough-colony Mycobacterium abscessus isolates. Altogether, we show that NTM aggregation is a controlled process that is governed by the relative availability of carbon and nitrogen for metabolism.

## Abstract (precis)

Free-living bacteria can assemble into multicellular structures called biofilms. Biofilms help bacteria tolerate multiple stresses, including antibiotics and the host immune system. Nontuberculous mycobacteria are a group of emerging opportunistic pathogens that utilize biofilms to adhere to household plumbing and showerheads and to avoid phagocytosis by host immune cells. Typically, bacteria regulate biofilm formation by controlling expression of adhesive structures to attach to surfaces and other bacterial cells. Mycobacteria harbor a unique cell wall built chiefly of long-chain mycolic acids that confers hydrophobicity and has been thought to cause constitutive aggregation in liquid media. Here we show that aggregation is instead a regulated process dictated by the balance of available carbon and nitrogen. Understanding that mycobacteria utilize metabolic cues to regulate the transition between planktonic and aggregated cells reveals an inroad to controlling biofilm formation through targeted therapeutics.

## INTRODUCTION

The adhesive biofilm matrix can serve as a physical barrier against external stresses such as desiccation and predation, can interact with and sequester antimicrobial agents, and can short-circuit phagocyte signaling (1–4). Additionally, the three-dimensional (3D) structure of biofilms creates chemical gradients across a cellular population (5–9), resulting in a spectrum of physiologies and metabolisms which, along with genetic diversification and stochastic differences in gene expression, give rise to substantial cell-to-cell heterogeneity (9–12). Heterogeneous bacterial communities demonstrate increased fitness compared to homogenous communities in a variety of models and experimental systems (11–13). Notably, most antibiotics target rapidly dividing bacteria, so slow-growing and dormant cells that develop in biofilms contribute to antibiotic tolerance (5, 10, 14–17).

Bacteria have evolved to enter and exit from the biofilm state in response to species- and strain-specific environmental signals. Cell-cell adhesion is a pivotal step in biofilm development in all bacteria, including nontuberculous mycobacteria (NTM) (18). Peculiarly, mycobacteria spontaneously aggregate under nearly all laboratory culture conditions, forming hydrophobic clumps in shaking cultures (19–22). This constitutive aggregation suggests either that mycobacteria express adhesive structures in response to signals that are very common in laboratory cultures or that they have adapted to always grow as aggregates in aqueous environments. The latter possibility has become the dominant paradigm, exemplified by the common addition of detergents such as Tween 80 to mycobacterial cultures to prevent clumping (20, 21, 23).

NTM are emerging pathogens that utilize various forms of aggregation for survival and persistence both in the host and in the nonhost environment (22, 24–27). From the baseline aggregated state, environmental parameters such as high iron trigger the maturation of NTM biofilms (18, 28). Biofilm formation is important for the ability of NTM to survive standard water decontamination protocols and to persist in household water systems (24, 29, 30). NTM can infect healthy adults after repeated exposure and are especially dangerous to immunocompromised populations and patients with lung disorders such as cystic fibrosis (CF) and chronic obstructive pulmonary disease (COPD) (29, 31–33). Infections with NTM can be very difficult to treat; Mycobacterium abscessus lung infections, in particular, require long courses of antibiotic cocktails that have limited efficacy and extensive adverse side effects (31, 34, 35). When M. abscessus acquires mutations that reduce expression of surface glycopeptidolipids (GPLs), it develops a rough-colony morphology and forms cords, rope-like structures in which the axis of each bacterial cell is parallel to the axis of the cord, in liquid culture (21, 36–39). Rough-colony isolates demonstrate increased pathogenicity in a zebrafish model and an enhanced ability to evade phagocytosis (22, 25, 26), indicating that the formation of multicellular structures by NTM is positively related to their sustained infection of hosts.

Because cell-cell adhesion is a requirement for biofilm formation and cording, a more thorough understanding of the regulatory pathways underpinning mycobacterial aggregation holds promise for combating NTM infections. In this study, we set out to understand whether and how aggregation is regulated in NTM. Toward this end, we developed an assay to quantify mycobacterial aggregation in liquid media under various nutritional conditions. Contrary to the conventional wisdom, we found that aggregation and dispersal are regulated processes in a variety of NTM, both pathogenic and nonpathogenic, dictated in large part by the relative availability of carbon and nitrogen.

## RESULTS

### Mycobacterial aggregates disperse as cultures age.

**FIG 1:**
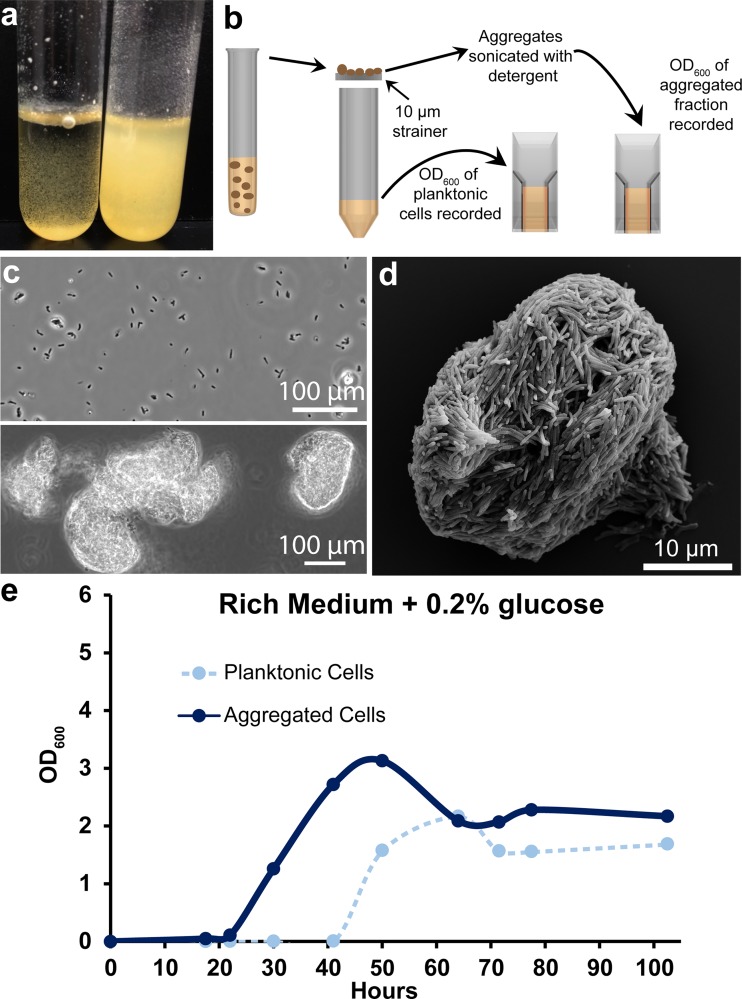
Quantification of mycobacterial aggregation/dispersal over time. (a) In rich medium plus 0.2% glucose, M. smegmatis grows as clumps at early time points (left tube, 30 h of growth). In older cultures, planktonic cells accumulate (right tube, 72 h of growth). (b) Cartoon depicting a method to separate and quantify aggregated and planktonic mycobacterial cells. (c) Phase-contrast micrograph showing the planktonic (top panel) and aggregated (bottom panel) fraction of a 72-h-old culture. The planktonic fraction is largely single cells and small clumps. Cells that are retained on the strainer (aggregated fraction) mostly exist as large clumps. (d) SEM of a representative M. smegmatis aggregate that was retained on the strainer after ∼30 h of growth in rich medium. (e) Aggregation curve of WT M. smegmatis grown in rich medium plus 0.2% glucose. Cells were harvested at each indicated time point and processed with the method outlined in panel b. Data are representative of *n* = 4 trials.

During routine culture in a rich medium with no detergent, the model NTM Mycobacterium smegmatis MC^2^155 grows as aggregated clumps. However, we noted that nonaggregated (planktonic) cells accumulated after ∼40 h of growth (Fig. 1a). We developed an assay to distinguish and quantify aggregated cells and planktonic cells over time. Briefly, culture replicates were harvested by passing an entire culture through a 10-μm cell strainer. The optical density at 600 nm (OD_600_) of cells that passed through the strainer (planktonic fraction) was immediately recorded. Aggregates that collected on the strainer were water bath sonicated in phosphate-buffered saline (PBS) plus 24.8% Tween 20, and the OD_600_ of the resultant suspension was recorded (Fig. 1b). Phase-contrast microscopy revealed that the planktonic fraction was composed mostly of single cells and small clusters of 2 to 4 cells (Fig. 1c). Scanning electron microscopy (SEM) of a representative aggregate revealed a densely packed structure (Fig. 1d). Performing this assay on M. smegmatis grown in rich medium plus 0.2% glucose revealed a decrease in the aggregate fraction concurrent with planktonic cell accumulation after ∼40 h of growth, suggesting a mechanism of controlled dispersal (Fig. 1e).

### Mutations in oligopeptide permease genes cause early dispersal.

**FIG 2:**
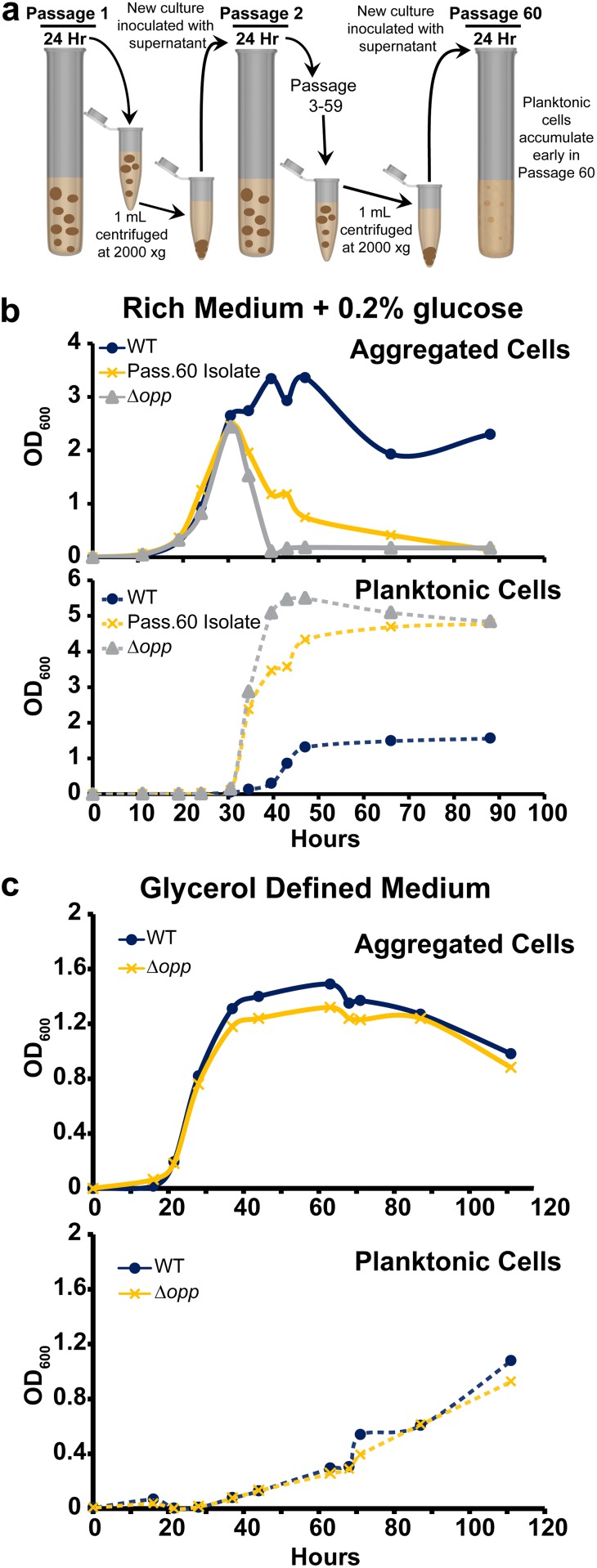
Mutations in an oligopeptide permease operon lead to early dispersal. (a) Cartoon depicting an evolution experiment to select for an M. smegmatis strain that disperses earlier than WT. (b) Aggregation curve of WT M. smegmatis, the passage 60 isolate, and the Δ*opp* mutant grown in rich medium plus 0.2% glucose. The top panel shows the aggregated fraction, and the bottom panel shows the planktonic fraction. Data are representative of *n* = 3 trials. (c) Aggregation curve of WT M. smegmatis and the Δ*opp* mutant grown in glycerol defined medium. The top panel shows the aggregated fraction, and the bottom panel shows the planktonic fraction. Data are representative of *n* = 2 trials.

To gain insight into the genetic regulation of M. smegmatis aggregation and dispersal, we designed an evolution experiment to select for mutants that disperse earlier than wild type (WT) in rich medium plus 0.2% glucose. Briefly, every 24 h 1 ml of a 5-ml culture was centrifuged at low speed to pellet aggregates. A new 5-ml culture was inoculated with 100 μl of the supernatant and grown for another 24 h (Fig. 2a). After 60 passages (roughly 575 doublings), planktonic cells visibly accumulated after 24 h of growth. Passage 60 was plated, and a single colony was selected and cultured. The passage 60 isolate displayed an early-dispersal phenotype compared to WT in rich medium plus 0.2% glucose (Fig. 2b). We sequenced the genomes of the passage 60 isolate, our WT strain (passage 0), and a passage 40 isolate that showed no early-dispersal phenotype (see Fig. S1A in the supplemental material). In total, the passage 40 isolate had 13 mutations compared to our passage 0 isolate, seven of which were in nontransposon open reading frames (ORFs). The passage 60 isolate had 11 mutations compared to our passage 0 isolate, nine of which were in nontransposon ORFs (Table 1).

**TABLE 1:** Nontransposon ORFs mutated in passage 40 and passage 60 isolates relative to the passage 0 isolate

Passage no. and gene^*d*^	Function	Mutation type	Mutation
Passage 40			
MSMEG_1808	SufE	Missense	Glu39Val
MSMEG_2148	HNH^*e*^ endonuclease domain-containing protein	Frameshift	Ser534fs^*a*^
MSMEG_5061	Extracellular solute binding protein	Missense	Ser249Pro
MSMEG_5808	Binding protein-dependent transporter	Missense	Arg117Cys
MSMEG_6397	Hypothetical protein	Missense	Ser21Pro
MSMEG_6430	Hypothetical protein	Missense	Thr371Lys
MSMEG_6821	NLP/P60 family protein	Missense	Gln2017Arg
Passage 60			
**MSMEG_0639**	**Oligopeptide transport ATP-binding protein OppF**	**Frameshift**	**Lys12fs**^*b*^
**MSMEG_0640**	**Oligopeptide transport ATP-binding protein OppD**	**Missense**	**Phe96Leu**
MSMEG_2148	HNH endonuclease domain-containing protein	Missense	Pro380Arg
MSMEG_2148	HNH endonuclease domain-containing protein	Frameshift	Ser534fs^*a*^
MSMEG_3677	Serine/threonine protein kinase	Silent	Val320Val
MSMEG_4217	DivIVA protein	Missense	Glu107Gly
MSMEG_5061	Extracellular solute binding protein	Frameshift	Glu225fs^*c*^
**MSMEG_5395**	**Sensor histidine kinase KdpD**	**Missense**	**Arg627Cys**
**MSMEG_6497**	**Hypothetical protein**	**Missense**	**His43Gln**

a MSMEG_2148 is 544 amino acids. Ser534 frameshift hypothetically replaces the 8 C-terminal amino acids with a different 23-amino-acid sequence.

b MSMEG_0639 is 336 amino acids. Lys12 frameshift hypothetically replaces the 325 C-terminal amino acids with a different 24-amino-acid sequence.

c MSMEG_5061 is 465 amino acids. Glu225 frameshift hypothetically replaces the 241 C-terminal acids with a different 236-amino acid-sequence.

d Bold text indicates the genes that were mutated in WT to test for aggregation defects.

e His-Asn-His (HNH).

10.1128/mBio.01715-19.1 FIG S1 The passage 40 isolate, Δ*kdpD* (MSMEG_5395), and Δ*MSMEG_6497* display no aggregation defects. (A) Aggregation curve of passage 0 (WT), 40, and 60 isolates in rich medium plus 0.2% glucose. The top panel shows the aggregated fraction, and the bottom panel shows the planktonic fraction. Data are representative of *n* = 2 trials. (B) Aggregation curve of WT, Δ*kdpD* (MSMEG_5395), and Δ*MSMEG_6497* in rich medium plus 0.2% glucose. The top panel shows the aggregated fraction, and the bottom panel shows the planktonic fraction. Data are representative of *n* = 2 trials. Download FIG S1, TIF file, 2.8 MB. Copyright © 2019 DePas et al. 2019 DePas et al. This content is distributed under the terms of the Creative Commons Attribution 4.0 International license.

To identify dispersal-related mutations, we narrowed our list of passage 60 candidate genes by discarding two genes that were also mutated in the passage 40 isolate (MSMEG_2148 and MSMEG_5061), one gene that acquired a silent mutation (MSMEG_3677), and *divIVA* (MSMEG_4217), because it is essential in M. smegmatis (40). We generated deletion mutants in a WT background of the four remaining candidates: *oppF*, *oppD*, *kdpD*, and the hypothetical gene MSEMG_6497. Because *oppF* and *oppD* code for two ATPase subunits associated with an oligopeptide permease (Opp) complex, we deleted the entire 5-gene *opp* operon (MSMEG_0643 to MSMEG_0639, termed Δ*opp*). While the Δ*kdpD* and Δ*MSMEG_6497* mutants showed no dispersal phenotype (Fig. S1B), the Δ*opp* mutant phenocopied the passage 60 isolate by displaying early dispersal (Fig. 2b). Complementation of the *opp* operon via the integration vector pMH94-*opp* restored aggregation in both Δ*opp* and the passage 60 isolate (Fig. S2) (41). Altogether, these results indicate that a functional oligopeptide permease system helps maintain aggregation in rich medium plus 0.2% glucose.

10.1128/mBio.01715-19.2 FIG S2 Complementation of the Δ*opp* mutant and the passage 60 isolate in rich medium plus 0.2% glucose. The left panels show aggregation curves of WT::pMH94 empty vector (EV), Δ*opp*::pMH94 EV, and Δ*opp*::pMH94-*opp* in rich medium plus 0.2% glucose and 5 μg/ml kanamycin. The right panels show aggregation curves of the same WT::pMH94 EV, passage 60 isolate::pMH94 EV, and passage 60 isolate::pMH94-*opp* in rich medium plus 0.2% glucose and 5 μg/ml kanamycin. The top panels show the aggregated fractions, and the bottom panels show the planktonic fractions. Data are representative of *n* = 2 trials. Download FIG S2, TIF file, 2.8 MB. Copyright © 2019 DePas et al. 2019 DePas et al. This content is distributed under the terms of the Creative Commons Attribution 4.0 International license.

The Opp complex imports oligopeptides for signaling and/or catabolism in multiple bacterial species (42, 43). Our rich medium contains tryptone and yeast extract, both of which are composed largely of oligopeptides, so we reasoned that (i) exogenous peptides themselves are a proaggregation signal, (ii) a self-produced peptide pheromone serves as a proaggregation signal, or (iii) metabolizing peptides as nutrients provides the cell with a proaggregation signal. To distinguish between these possibilities, we grew WT and Δ*opp* strains in a defined, peptide-free glycerol medium. If exogenous peptides are necessary for aggregation (i), neither WT nor Δ*opp* should aggregate in the peptide-free medium; if a self-produced pheromone is required for aggregation (ii), WT should aggregate but Δ*opp* should be defective; and if peptides are used as a nutrient that provides a proaggregation signal (iii), providing the cells with alternative carbon and nitrogen sources should bypass the need for peptide import and both strains should aggregate. WT and Δ*opp* maintained aggregation to similar degrees in glycerol defined medium (Fig. 2c), suggesting that the Opp complex promotes aggregation in rich medium by increasing cells’ access to the peptide nutrient sources.

### Carbon availability dictates M. smegmatis aggregation and dispersal.

**FIG 3:**
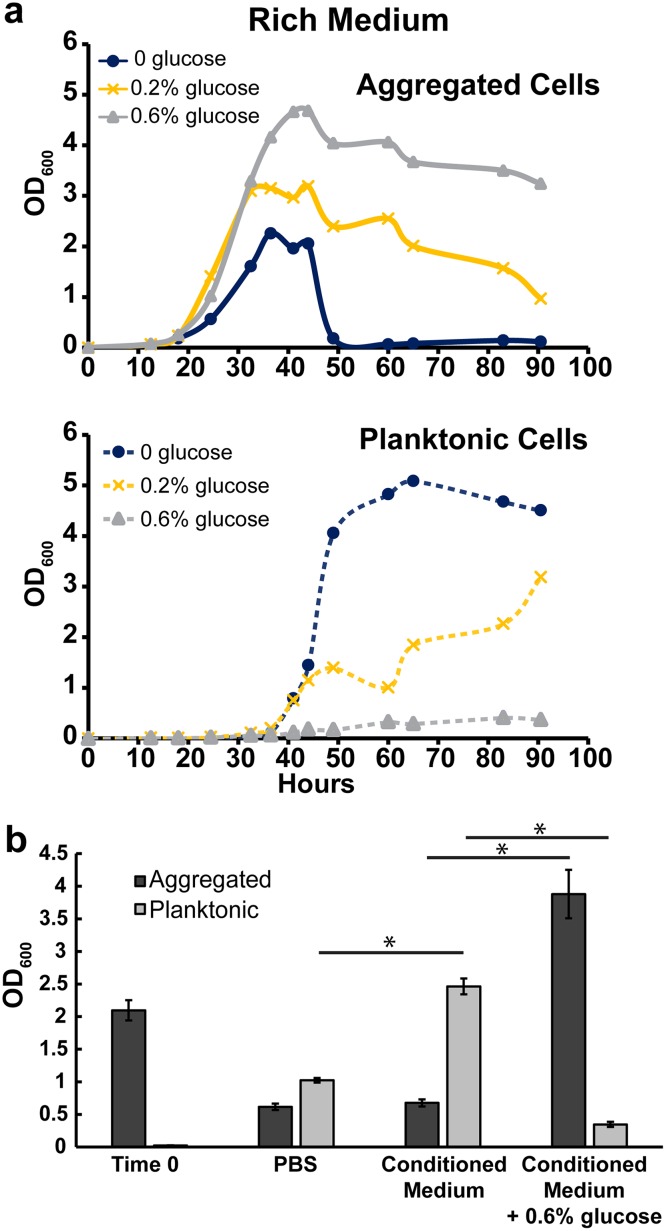
Carbon depletion leads to dispersal. (a) Aggregation curve of WT M. smegmatis in rich medium with no glucose, 0.2% glucose, or 0.6% glucose. The top panel shows the aggregated fraction, and the bottom panel shows the planktonic fraction. Data are representative of *n* = 3 trials. (b) Aggregates harvested from 48-h-old rich medium cultures (Time 0) were resuspended in conditioned medium (filter sterilized from 52-h-old rich medium cultures), PBS, or conditioned medium plus 0.6% glucose and grown for 12 h. Each bar is an average from biological triplicates, and error bars represent standard deviations. Asterisks represents *P* < 0.05 by the Student *t* test.

Because the Δ*opp* mutant’s aggregation deficiency in rich medium plus 0.2% glucose appeared to be due to a defect in nutrient uptake, we tested whether nonpeptide carbon supplementation could complement this defect. Indeed, glucose addition prolonged aggregation in both WT and Δ*opp*, suggesting that carbon starvation is a signal for dispersal (Fig. 3a and Fig. S3A). Because of the utility of being able to measure nearly complete dispersal, rich medium experiments going forward contain no glucose unless otherwise noted. If carbon starvation leads to aggregate dispersal, we would predict that either carbon-free buffer or carbon-depleted medium should be sufficient to induce dispersal. We therefore resuspended WT aggregates (grown in rich medium for 48 h) in either PBS or conditioned medium from 52-h-old cultures. After 12 h, we harvested and quantified aggregated and planktonic populations (Fig. 3b). Aggregates decreased to similar degrees in both conditioned medium and PBS (Fig. 3b). Furthermore, when 0.6% glucose was added to conditioned medium, dispersal was largely prevented (Fig. 3b). Unexpectedly, when aggregates were resuspended in conditioned medium, planktonic cells accumulated to a significantly higher extent than in PBS (Fig. 3b). This result indicated that, instead of growth as aggregates and subsequent dispersal, there may be a window of time in a rich medium culture wherein nutrient conditions favor growth as planktonic cells.

10.1128/mBio.01715-19.3 FIG S3 Response of the Δ*opp* mutant to glucose or ammonium. (A) Aggregation curve of Δ*opp* in rich medium with no glucose, 0.2% glucose, or 0.6% glucose. The top panel shows the aggregated fraction, and the bottom panel shows the planktonic fraction. Data are representative of *n* = 2 trials. (B) Aggregation curve of Δ*opp* in rich medium with no glucose with no NH_4_Cl, 25 mM NH_4_Cl, or 75 mM NH_4_Cl. The top panel shows the aggregated fraction, and the bottom panel shows the planktonic fraction. Data are representative of *n* = 2 trials. Download FIG S3, TIF file, 0.4 MB. Copyright © 2019 DePas et al. 2019 DePas et al. This content is distributed under the terms of the Creative Commons Attribution 4.0 International license.

### Low C/N ratio drives growth as planktonic cells.

**FIG 4:**
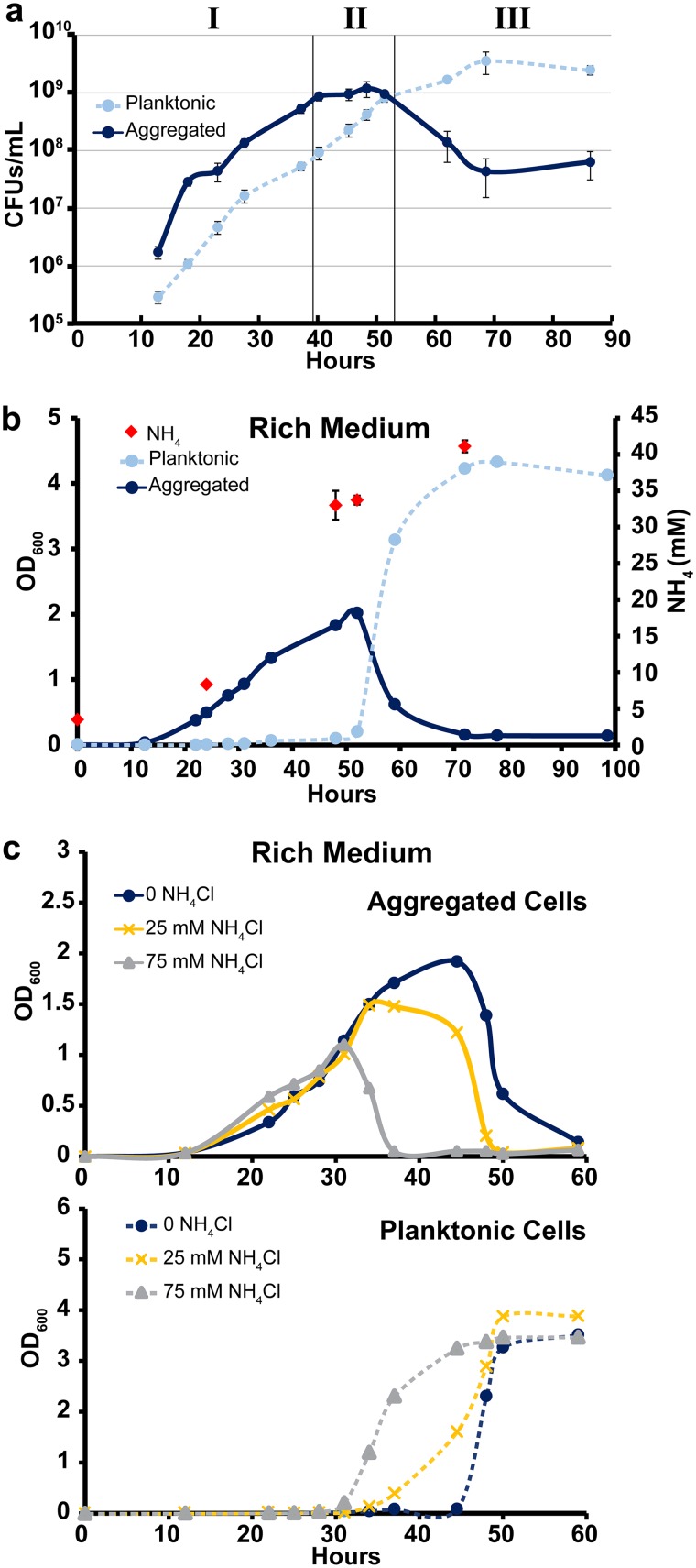
Low C/N availability favors growth as planktonic cells. (a) CFUs per milliliter for WT M. smegmatis grown in rich medium (no glucose). Each data point is the average from biological triplicates, and error bars represent standard deviations. Roman numerals denote three phases of growth as described in the text. (b) Aggregation curve of WT M. smegmatis in rich medium (no glucose). At indicated time points, three additional cultures were harvested for NH_4_ IC measurements. Each NH_4_ data point is an average from biological triplicates, and error bars represent standard deviations. Aggregation curve data are representative of *n* = 5 trials. (c) Aggregation curve of WT M. smegmatis in rich medium (no glucose) with no NH_4_Cl, 25 mM NH_4_Cl, or 75 mM NH_4_Cl. The top panel shows the aggregated fraction, and the bottom panel shows the planktonic fraction. Data are representative of *n* = 3 trials.

Because the OD_600_ has a limited range in which it can accurately measure cell density, we measured CFUs/ml of both aggregated and planktonic fractions over time in rich medium (Fig. 4a). Aggregates were dispersed by short water bath sonication in rich medium with Tween 80 and Tween 20 prior to plating (Fig. S4). This experiment revealed three distinct phases of growth. In phase I (∼0 to 40 h), the fractions grow at similar rates with the aggregated fraction outnumbering the planktonic fraction by roughly 10-fold. In phase II (∼40 to 53 h), planktonic cells continue growing while aggregated fraction growth ceases. Then, in phase III (at ∼53 h onward), aggregates disperse and the planktonic fraction enters stationary phase (Fig. 4a). Our results from Fig. 3 suggest that carbon excess and depletion drive growth as aggregates and aggregate dispersal, respectively. Therefore, we sought to characterize the phase II culture conditions that favored planktonic cell growth.

10.1128/mBio.01715-19.4 FIG S4 Homogenizing aggregates for CFU counts. Picture showing aggregated M. smegmatis culture grown in rich medium before (left tube) and after (right tube) water bath sonication in rich medium plus 0.05% Tween 80 plus 2% Tween 20 to homogenize aggregates for CFU counts. Download FIG S4, TIF file, 0.9 MB. Copyright © 2019 DePas et al. 2019 DePas et al. This content is distributed under the terms of the Creative Commons Attribution 4.0 International license.

One well-described side effect of bacterial growth on peptides is the release of excess ammonium into the medium (44). Indeed, ammonium levels increased as our cultures aged, reaching ∼33 mM at 48 h (Fig. 4b). To test whether ammonium facilitated growth as planktonic cells, we added excess NH_4_Cl to starting cultures and tracked aggregation. Ammonium addition led to earlier accumulation of planktonic cells and reduced aggregation (Fig. 4c and Fig. S3B). To test whether salts have a general effect on aggregation, we added 75 mM NaCl to WT cultures. NaCl did not affect aggregation kinetics, indicating that ammonium specifically favors planktonic growth (Fig. S5). If the high ammonium concentration in conditioned medium favors growth as planktonic cells, it is notable that adding excess carbon to conditioned medium shifts the population back toward growth as aggregates (Fig. 3b). Altogether, these results are consistent with a model wherein carbon-replete conditions favor growth as aggregates, high-nitrogen (relative to carbon) conditions favor growth as planktonic cells, and carbon depletion leads to aggregate dispersal.

10.1128/mBio.01715-19.5 FIG S5 NaCl does not affect aggregation. Aggregation curve of WT in rich medium (no glucose) with or without 75 mM NaCl. The top panel shows the aggregated fraction and the bottom panel shows the planktonic fraction. Data are representative of *n* = 3 trials. Download FIG S5, TIF file, 1.2 MB. Copyright © 2019 DePas et al. 2019 DePas et al. This content is distributed under the terms of the Creative Commons Attribution 4.0 International license.

### Defined medium designed for growth as aggregated or planktonic cells.

**FIG 5:**
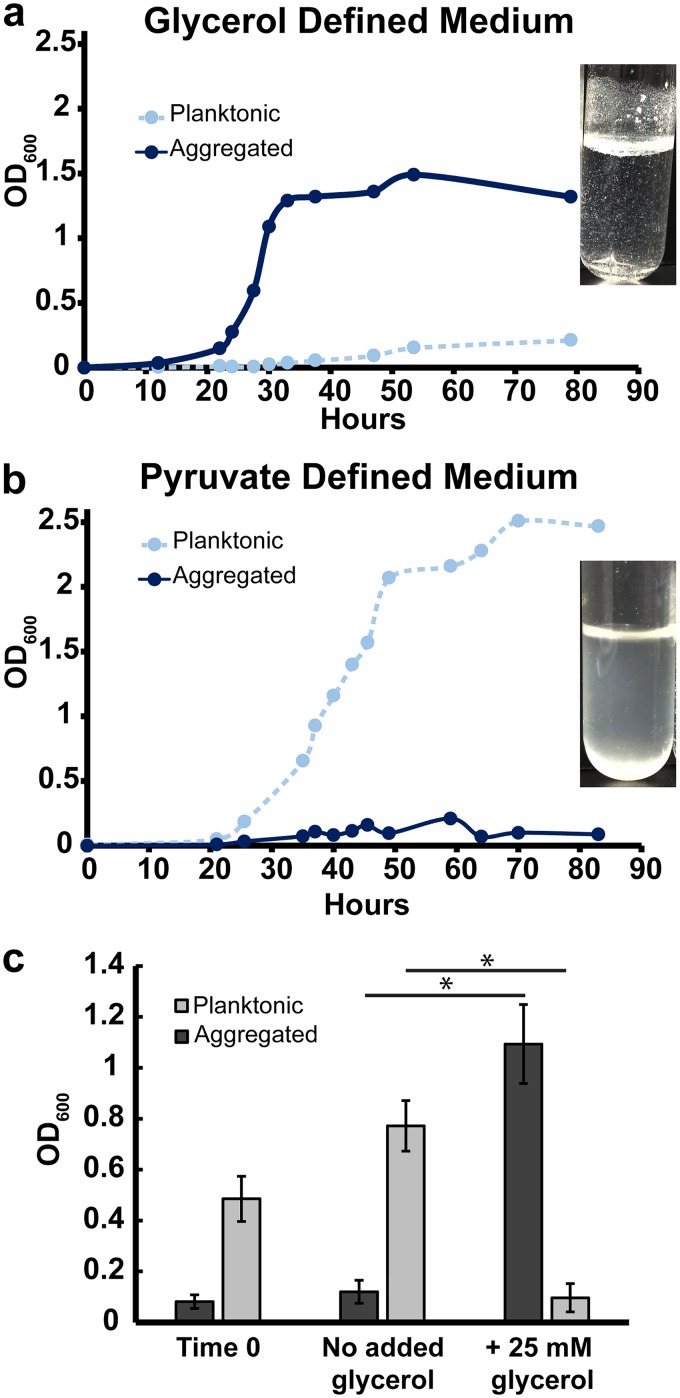
Defined medium designed to favor growth as aggregates or planktonic cells. (a) Aggregation curve of WT M. smegmatis in glycerol defined medium. Culture image was taken after 27 h of growth. Data are representative of *n* = 4 trials. (b) Aggregation curve of WT M. smegmatis in pyruvate defined medium. Culture image was taken after 34 h of growth. Data are representative of *n* = 4 trials. (c) WT M. smegmatis was grown in pyruvate plus NH_4_Cl defined medium for 34 h (Time 0). Glycerol was then added to 25 mM, and cultures were incubated for 6 more hours before harvesting. Bars represent biological triplicates, and error bars represent standard deviations. Asterisks represent *P* < 0.05 by the Student t test.

To test whether M. smegmatis is able to grow as planktonic cells at low C/N ratios, we designed defined medium to supply the bacteria with either replete carbon and low nitrogen (high C/N availability) or replete nitrogen and low carbon (low C/N availability). To grow M. smegmatis with high C/N availability, we used glycerol as the main carbon source, glutamate as the main nitrogen source, and no ammonium (117 mM carbon, 5.5 mM nitrogen; C/N of the medium = 21.4). Glycerol is commonly supplied to mycobacteria because it supports fast growth and, as a small (three-carbon) uncharged molecule, can presumably passively diffuse across the mycolic acid barrier (45). Indeed, growth on glycerol floods most central metabolite pools compared to growth on other carbon sources in Mycobacterium tuberculosis (46). To generate low C/N availability, we used a charged three-carbon compound, pyruvate, as the main carbon source and added 20 mM NH_4_Cl in addition to glutamate as the nitrogen source (117 mM carbon, 25.5 mM nitrogen; C/N of the medium = 4.58). In some bacteria, the relative availability of carbon and nitrogen sources can be reflected in total C/N content of the cell (47, 48). Therefore, to assess whether our medium was effectively providing high or low C/N availability, we directly measured the ratio of cellular carbon to cellular nitrogen (by mass) of M. smegmatis grown in either pyruvate or glycerol medium when the total OD_600_ was between 0.5 and 0.7. As predicted, M. smegmatis grown on glycerol had a C/N ratio of 6.95 (standard deviation [SD], 0.85) and on pyruvate plus NH_4_Cl had a C/N ratio of 5.02 (SD, 0.31; *P* = 0.005). Consistent with our hypothesis, M. smegmatis grew mostly as aggregates on glycerol and grew mostly as planktonic cells on pyruvate (Fig. 5a and b).

The C/N ratio in natural environments such as soil affects bacterial diversity and growth and is often tuned in order to favor desired bacterial metabolisms in industrial settings (49, 50). It is therefore notable that even when grown in pyruvate defined medium with no ammonium (117 mM carbon, 5.5 mM nitrogen; C/N of the medium = 21.4, equal to glycerol defined medium), M. smegmatis had a relatively low cellular C/N ratio of 5.23 (SD, 0.38; *P* = 0.01 compared to glycerol-grown cells) and grew as mostly planktonic cells (Fig. S6). These results reinforce that the form of available nutrients, and not just total carbon and nitrogen in an environment, can impact a cell’s C/N status and dependent phenotypes.

10.1128/mBio.01715-19.6 FIG S6 Growth as planktonic cells in pyruvate defined medium with no NH_4_Cl. Aggregation curve of WT in pyruvate defined medium (with no N_4_Cl). Data are representative of *n* = 3 trials. Download FIG S6, TIF file, 0.7 MB. Copyright © 2019 DePas et al. 2019 DePas et al. This content is distributed under the terms of the Creative Commons Attribution 4.0 International license.

Last, we leveraged our pyruvate defined medium to test whether planktonic cells can transition to aggregates. Planktonic M. smegmatis was grown for 36 h in pyruvate plus NH_4_Cl defined medium before addition of 0 or 25 mM glycerol. By 6 h after glycerol addition, the majority of the planktonic population had aggregated (Fig. 5c), further demonstrating that the aggregation state is dynamic and dependent on the ratio of available C to N.

### Liquid aggregation state correlates with colony biofilm morphology.

Liquid aggregation is indicative of cell-cell adhesion, a necessary step in the development of a mature mycobacterial biofilm (18). We therefore hypothesized that liquid aggregation dynamics would correlate with biofilm formation. Using the colony morphology model of biofilm development, we observed that WT M. smegmatis on rich medium agar plates wrinkled and then smoothed out over time (Fig. S7). When grown on glycerol defined medium agar plates, M. smegmatis formed wrinkled colonies that did not smooth out in the same time frame, paralleling our liquid aggregation assays and demonstrating that the aggregation state of NTM in liquid medium can predict biofilm-forming capacity (Fig. S7).

10.1128/mBio.01715-19.7 FIG S7 Colony biofilm development mirrors liquid aggregation dynamics. Cultures of WT M. smegmatis grown in rich medium were diluted to an OD_600_ of 1, and 2-μl spots were plated on rich medium or glycerol defined medium agar plates (1.7% agar). Plates were incubated at 37°C with Parafilm, and pictures were taken every day. Download FIG S7, TIF file, 2.6 MB. Copyright © 2019 DePas et al. 2019 DePas et al. This content is distributed under the terms of the Creative Commons Attribution 4.0 International license.

### C/N-dependent aggregation regulation is common among NTM.

**FIG 6:**
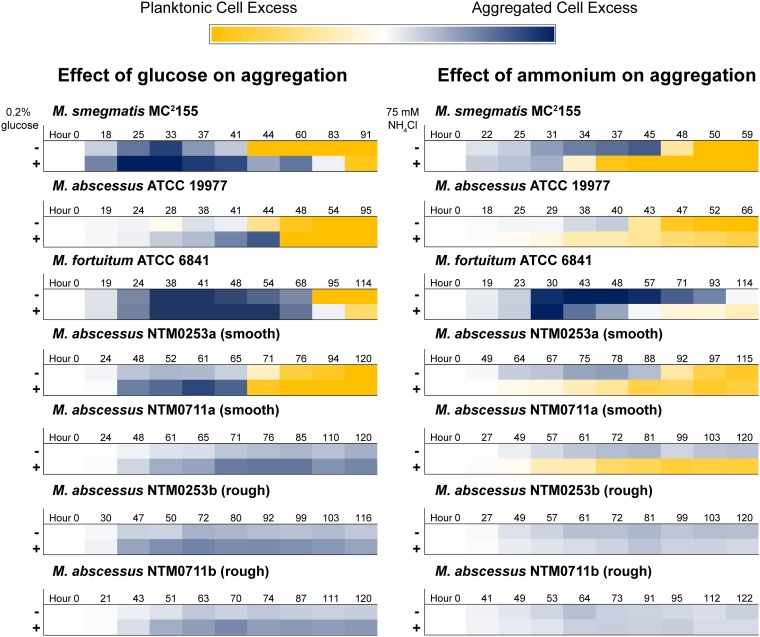
C/N regulation of aggregation/dispersal is common among NTM. Aggregation curves in rich medium with or without 0.2% glucose (left column) or rich medium with or without 75 mM NH_4_Cl (right column) were recorded for indicated strains. Ten time points were selected from each curve to span the entire time course. The OD_600_ value of the planktonic fraction was multiplied by −1, and then the OD_600_ values of the two fractions were added together. The darkest blue color corresponds to sums of 2.5 or greater, and the darkest yellow color corresponds to sums of −2.5 or less. Times are rounded up to the nearest hour. Data are representative of at least *n* = 2 trials. The M. smegmatis heat maps represent aggregation curves shown in Fig. 3a and Fig. 4c. The aggregation curves from which the other heat maps were derived are included in Fig. S9.

To determine whether C/N regulation of aggregation is conserved among clinically relevant NTM, we grew type strains of M. abscessus and Mycobacterium fortuitum along with four M. abscessus subsp. *abscessus* clinical isolates from CF patients in rich medium and tracked aggregation kinetics and aggregate morphology. As expected, microscopic inspection of our strains demonstrated that our two rough M. abscessus isolates formed distinct cords in rich medium while the other strains formed more generic aggregates (Fig. S8) (22, 39). Both type strains and one smooth-colony clinical isolate accumulated planktonic cells at later culture time points, with glucose addition increasing total aggregation and ammonium addition favoring growth as planktonic cells (Fig. 6 and Fig. S9). Neither rough-colony M. abscessus isolate accumulated planktonic cells, even after addition of ammonium. In contrast, the smooth-colony isolate that did not disperse in rich medium grew solely as planktonic cells when provided with supplemental ammonium. Altogether, our results indicate that C/N balance is a common regulator of NTM aggregation, with corded rough-colony clinical isolates being a possible exception.

10.1128/mBio.01715-19.8 FIG S8 Aggregate morphologies during liquid growth. Representative images of aggregates grown in rich medium, harvested, and imaged in Lab-Tek II chambered coverglass dishes. Download FIG S8, TIF file, 2.0 MB. Copyright © 2019 DePas et al. 2019 DePas et al. This content is distributed under the terms of the Creative Commons Attribution 4.0 International license.

10.1128/mBio.01715-19.9 FIG S9 C/N regulation of aggregation/dispersal is common among NTM. Aggregation curves of M. abscessus ATCC 19977, M. fortuitum ATCC 6841, two smooth-colony M. abscessus subsp. *abscessus* isolates (NTM0253a and NTM0711a), and two rough-colony M. abscessus subsp. *abscessus* isolates (NTM0253b and NTM0711b). Strains were grown in rich medium with or without 0.2% glucose (top row in each panel) and in rich medium (no glucose) with or without 75 mM NH_4_Cl (bottom row in each panel). Data are representative of at least *n* = 2 trials. Download FIG S9, TIF file, 1.0 MB. Copyright © 2019 DePas et al. 2019 DePas et al. This content is distributed under the terms of the Creative Commons Attribution 4.0 International license.

## DISCUSSION

The role of biofilm formation in rendering bacteria recalcitrant to antibiotics and immune killing provides motivation to develop novel antibiofilm strategies. However, because bacteria have evolved to occupy and form biofilms in diverse ecological niches, the regulatory pathways and physical components that govern biofilm formation differ significantly between species. As such, a species-specific, in-depth understanding of how cells sense and respond to their environment by aggregating under certain conditions, and growing as planktonic cells under others, is essential in order to control bacterial biofilm formation for any specific pathogen. In this work, we have found a role for C/N balance in dictating the transition between planktonic and aggregated states in NTM.

Understanding the environmental niches in which NTM have evolved can lend context to our finding that C/N balance controls aggregation state. NTM are nonmotile saprophytes that are common residents of soil and waterways (27, 35, 51). In soil, carbon is most often the limiting nutrient for bacterial growth (52, 53). At a low C/N ratio, our data suggest that NTM could exist at least partly as planktonic cells. As water flow is a major factor in determining movement of bacteria through soil (54, 55), NTM in this state might be susceptible to water-mediated transport to another region of the rhizosphere (potentially containing more carbon). Larger bacterial cell sizes correlate with decreased movement through soil (56). Therefore, if NTM were growing as aggregates under carbon-rich conditions, we would expect them to be less likely to be washed away into potentially more carbon-depleted regions. While speculative, this natural ecological context motivates us to consider how mycobacteria might sense the C/N balance in their environment and control their aggregation state accordingly.

It is well appreciated that carbon and nitrogen availability dictate the metabolic and growth capacity of a cell (57, 58), and bacteria are able to coordinate carbon and nitrogen metabolism through a variety of means (59). The cellular C/N ratio provides a rough estimate of the cell’s C/N status, but it is not a parameter that a cell can directly sense. How then do mycobacteria translate C/N availability to aggregation? Our data show that no one carbon source is necessary to drive aggregation. Interestingly, by responding to flux through a metabolic pathway, a cell can integrate the signal from multiple inputs without needing to measure each one specifically (60). It thus seems possible that mycobacteria sense and respond to flux-dependent metabolites—molecules whose intracellular pools correlate with flux through specific metabolic pathways, such as fructose-1,6-bisphosphate (FBP), the levels of which correlate with glycolytic flux (60, 61), or 2-oxoglutarate (2OG), the levels of which correlate to flux through the tricarboxylic acid (TCA) cycle (60, 62). Alternatively, or in addition, two-component systems might mediate the translation between metabolite availability and aggregation. Uncovering the pathways through which NTM achieve aggregation control is a priority for future work.

Regardless of the signal transduction mechanism, a surface adhesin must mediate the aggregation phenotype. Like many members of the *Corynebacteriales* order, mycobacteria produce a mycomembrane: a cell wall composed of peptidoglycan, arabinogalactan covalently linked to an inner leaflet of long-chain mycolic acids, and an outer layer of extractable lipids, lipoglycans, and proteins (63, 64). As such, the mycobacterial cell wall is unusually lipid rich (65, 66). A lipid-rich cell wall fits the longstanding observation that mycobacteria clump together into hydrophobic aggregates; in his original description of M. tuberculosis in 1882, Robert Koch noted that the bacteria “…ordinarily form small groups of cells which are pressed together and arranged in bundles” (67). Clumping is now recognized as a ubiquitous feature of mycobacteria, and as clumps are hydrophobic, detergents such as Tween 80 are almost universally added to mycobacterial cultures to favor growth as dispersed cells (22, 23, 68).

Inherent to the chemical intuition linking a lipid-rich cell wall and spontaneous clumping is the assumption that mycobacteria display a constitutively hydrophobic cell surface. Yet, several studies of the mycomembrane composition challenge this dogma. Mycolic acid chain length affects aggregation (69), and M. smegmatis can regulate mycolic acid chain length in response to environmental factors (70, 71). Additionally, genes involved in the biosynthesis and glycosylation of GPLs in M. smegmatis, Mycobacterium avium, and M. abscessus affect aggregation and cell surface hydrophobicity (37, 72–74). GPL production and glycosylation are also regulated by chemical signals (73, 75). In addition to providing evidence that mycobacteria can dynamically regulate cell envelope composition and surface hydrophobicity, these studies provide candidate adhesins that could be effectors of C/N-driven aggregation regulation.

Finally, the fact that NTM regulate aggregation has potentially important biomedical relevance. New treatments are needed to combat NTM infections, such as those caused by M. abscessus, which is notoriously difficult to eradicate. It is noteworthy that the corded, rough-colony isolates of M. abscessus subsp. *abscessus* do not disperse in rich medium. Because rough M. abscessus isolates are typically the result of mutations that reduce GPL production (36–38), we might hypothesize that C/N regulation is linked to GPL production or modification, which directly impacts the aggregation state. It is worth exploring whether nodes along such a pathway could be identified and exploited as new targets for biofilm control. The rising threat of NTM infections, particularly to susceptible communities such as CF patients, as well as the correlation between increased aggregation and virulence, lends motivation to further probe the mechanisms of aggregation and dispersal in these pathogens (22, 25, 34, 35).

## MATERIALS AND METHODS

### Strains, growth conditions, and cloning.

Strains, plasmids, and primers used in this study are listed in Table S1 in the supplemental material. The rich medium used in this study was TYEM (10 g tryptone, 5 g yeast extract/liter, plus 2 mM MgSO_4_). Where noted, filter-sterilized glucose or NH_4_Cl was added as supplements to autoclaved TYEM. For routine culturing of mycobacteria, bacteria were grown in TYEM for ∼50 to 70 h, at which time cultures were passed through 10-μm strainers (from pluriSelect; catalog no. 43-50010-03) and planktonic cells were collected and processed. The exception was rough M. abscessus isolates NTM0253b and NTM0711b, which were cultured in TYEM plus 0.05% Tween 80. Our defined medium was modified M63—13.6 g KH_2_PO_4_ was dissolved in 500 ml Nanopure H_2_O and the pH was adjusted to 7.0 via addition of KOH. This 2× stock was filter sterilized and diluted to 1× with Nanopure H_2_O while adding filter-sterilized supplements: MgSO_4_ to 1 mM, FeSO_4_ to 10 μM, SL-10 trace metal solution to 1×, proline to 0.5 mM, sodium glutamate to 5 mM, NH_4_Cl to 20 mM (when noted), and either glycerol to 30 mM or sodium pyruvate to 30 mM. Mutants of M. smegmatis MC^2^155 were made via recombineering as described previously with minor alterations (76). Briefly, M. smegmatis transformed with pJV53 was grown in TYEM plus 0.05% Tween 80 plus 25 μg/ml kanamycin until it reached an OD_600_ of 0.4 to 0.5. Acetamide was added to 0.2%, and cells were incubated for 3 h with shaking at 250 rpm at 37°C. Cells were then made electrocompetent by serial washes with chilled 10% glycerol (1/2, 1/10, 1/20, and 1/100 original volume) with centrifugation at 4,000 × *g* for 10 min at 4°C between washes. One hundred microliters of the cell mixture was then electroporated with 200 ng of linear DNA encoding a gentamicin resistance cassette (PCR amplified from plasmid pMQ30) flanked by 400 to 500 bp of sequence upstream and downstream of the target genes. Flanking regions were PCR amplified from WT M. smegmatis colonies, and Gibson assembly was utilized to combine flanking regions with the gentamicin resistance cassette. After mutagenesis, mutant strains were cured of pJV53 by passaging on TYEM with no antibiotics 3 to 7 times until they were verified as kanamycin sensitive. The *opp* operon was cloned from WT M. smegmatis into pMH94 using the XbaI site. pMH94 was integrated into M. smegmatis via electroporation as described previously (41).

10.1128/mBio.01715-19.10 TABLE S1 Strains, plasmids, and primers used in this study and their original source. Download Table S1, DOCX file, 0.02 MB. Copyright © 2019 DePas et al. 2019 DePas et al. This content is distributed under the terms of the Creative Commons Attribution 4.0 International license.

### Light microscopy and SEM.

For light microscopy shown in Fig. 1, samples were loaded onto Tekdon poly-l-lysine-coated slides and phase-contrast images were acquired on a Zeiss AxioObserver.A1 using a 40× 1.3-numerical aperture (NA) oil immersion objective. Images in Fig. S8 in the supplemental material were acquired with the T-PMT detector of a Zeiss LSM 800 confocal microscope and a 10× 0.45-NA objective. For SEM, WT M. smegmatis was grown in rich medium for 24 h, at which point the culture was passed through a 10-μm strainer and washed with PBS. Aggregates that collected on the strainer were fixed in 4% paraformaldehyde (PFA) for 2 h at room temperature, washed twice with PBS, and fixed in 1% OsO_4_ for 1 h at room temperature. After two more rinses with PBS, aggregates were dehydrated in an ethanol series, with 10-min incubations in 50%, 70%, 90%, 95%, and 100% ethanol, and a final incubation in 100% ethanol for 1 h. Samples were then incubated in a 1:2 solution of hexamethyldisilazane (HMDS)-ethanol for 20 min and a 2:1 solution of HMDS-ethanol for 20 min, followed by two incubations in 100% HMDS for 20 min each. Samples were then loaded onto silicon wafers, air dried, and attached to imaging stubs with conductive tape. Samples were sputter coated with 10 nm of palladium and imaged on a Zeiss 1550VP field emission SEM using an SE2 detector.

### Aggregation assays.

Medium for aggregation assays was prepared in flasks and inoculated with the indicated strain of bacteria to an OD_600_ of 0.01. After mixing, 5-ml aliquots were pipetted into brand-new borosilicate disposable culture tubes. These culture replicates were incubated at 37°C while being shaken at 250 rpm. At indicated time points, a single culture replicate was harvested by pouring the entire culture through a 10-μm strainer. Culture that passed through the strainer was designated the planktonic cell fraction, and the OD_600_ was immediately recorded. The original culture tube was washed with 5 ml of PBS, which was then poured over the aggregate fraction to remove residual planktonic cells. Aggregates that remained on the strainer were resuspended in 4.5 ml PBS plus 6% Tween 20 and poured back in the original culture tube. Five hundred microliters of Tween 20 was added for a final volume of 5 ml and a final Tween 20 concentration of 28.5%. Aggregate fractions were then water bath sonicated until no visible clumps remained, and the OD_600_ of the aggregate fraction was recorded. For CFU counts, a slightly modified protocol was employed for the aggregate fraction. Instead of PBS, aggregates were resuspended in TYEM plus 0.05% Tween 80, to which 100 μl of autoclave-sterilized Tween 20 was added. Aggregates were then water bath sonicated until no clumps were visible. Both planktonic and aggregate fractions were then serially diluted in TYEM plus 0.05% Tween 80, and serial dilutions spanning 7 orders of magnitude were plated on TYEM agar plates as 10-μl drips. Plates were incubated at 37°C for ∼2 days, and colonies were counted at the appropriate dilution. Conditioned medium was prepared by centrifuging 52-h-old cultures and filtering the supernatant through an 0.2-μm filter. For conditioned medium experiments, three 48-h-old cultures were pooled by passing them through a single 10-μm strainer. Aggregates were washed with 5 ml of PBS and then resuspended in 15 ml of conditioned medium (or PBS as indicated). Five-milliliter aliquots were partitioned into three culture tubes, and after 12 h of shaking at 37°C, aggregates and planktonic cells were separated and quantified.

### Evolution experiment/sequencing.

WT M. smegmatis was inoculated into TYEM plus 0.2% glucose. After 24 h, 1 ml of culture was centrifuged for 1 min at 2,000 × *g*. One hundred microliters of supernatant was inoculated into a new TYEM plus 0.2% glucose culture. The process was repeated every 24 h. After 60 passages, planktonic cells were visibly accumulating at 24 h. This culture was plated on TYEM agar plates, and a single colony was selected as the passage 60 isolate. Along with an isolate from passage 0 and passage 40, this strain was grown to mid-exponential phase, and DNA was extracted as described previously (77). DNA was fragmented using the NEBNext dsDNA Fragmentase (New England Biolabs, Ipswich, MA) according to the manufacturer’s instructions. Briefly, 1 μg of passage 0 and passage 40 DNA and 725 ng of passage 60 DNA were treated with Fragmentase for 15 min in order to achieve an acceptable size distribution, which was assessed using a high-sensitivity DNA chip on a Bioanalyzer instrument (Agilent). Libraries for sequencing were prepared using the NEBNext DNA library prep kit according to instructions, which included end repair of the fragments, deoxyribosyladenine (dA) tailing, and ligation to adaptors. Each sample was PCR amplified with a universal primer and a unique bar-coded primer, using 12 amplification cycles. Final libraries were verified using a Bioanalyzer high-sensitivity DNA chip and quantified using the Qubit fluorimeter and double-stranded DNA (dsDNA) dye (Invitrogen). Sequencing was performed by the Millard and Muriel Jacobs Genetics and Genomics Laboratory at the California Institute of Technology using the Illumina HiSeq 2500 platform. Approximately 15 million single reads of 50 bp each were collected for each sample. Base-calling and demultiplexing were performed by the Illumina HiSeq Control Software (HCS; version 2.0). The resulting FASTQ files were concatenated into one file per sample and filtered and trimmed by quality score per base using the Trimmomatic software package with the following parameters: LEADING:27 TRAILING:27 SLIDINGWINDOW:4:20 MINLEN:35 (78). Surviving reads were mapped to the Mycobacterium smegmatis strain MC^2^155 genome (gi|118467340|ref|NC_008596.1) using bwa (version 0.7.12) (79), and sorted and converted to binary format using SAMtools (version) (80). Tools from the Genome Analysis Tool Kit (GATK; version 2.7-4-g6f46d11) (81) were used to call single nucleotide polymorphism (SNPs) and small insertions and deletions relative to the reference genome as follows. First, duplicate reads were identified and marked using the MarkDuplicates tool. Next, putative insertions and deletions were identified using the RealignerTargetCreator tool, and reads surrounding them were realigned using the IndelRealigner tool. Finally, putative variants relative to the reference genome were called using the UnifiedGenotyper tool. One hundred forty-four variant regions were confidently identified in the passage 0 sample, 153 variant regions were identified in the passage 40 sample, and 154 variant regions were identified in the passage 60 sample. Most of these variations were common to all three samples and were not considered further. For mutations of interest, the effects on protein coding sequence were predicted using the SnpEff tool (version SnpEff 4.3t) (82). Nontransposon ORFs mutated in the passage 40 and passage 60 isolates relative to the passage 0 isolate are listed in Table 1.

### Ammonium measurements.

At the time points indicated, 1 ml of culture was centrifuged at 16,000 × *g* at room temperature for 1 min to pellet cells. Supernatants were filter sterilized through a 0.2-μm syringe filter and diluted 1:40 in Nanopure H_2_O. Parallel ion chromatography (IC) systems operated simultaneously (Dionex ICS 200; Environmental Analysis Center, Caltech) were used to measure ammonium. A single autosampler (Dionex AS 40) loaded both systems’ sample loops serially. The 5-μl sample loop on the anion IC system was loaded first, followed by a 5-μl sample loop on the cation IC system. Both columns and both detectors were maintained at 30°C. Anionic components in the sample were resolved using an AS-19 separator (2- by 250-mm) column protected by an AG-19 guard (2 by 50 mm). A hydroxide gradient was produced using a potassium hydroxide eluent generator cartridge and pumped at 0.25 ml/min. The gradient began with a 10 mM hold for 10 min and increased linearly to 58 mM at 25 min, remaining at 58 mM until the end of data acquisition at 32 min. Seven minutes was allowed between analyses to return the column to initial conditions. Anions were detected at neutral pH using an AERS-500 2-mm suppressor (Thermo) operated in eluent recycle mode with an applied current of 30 mA and conductivity detection cell maintained at 35°C. A carbonate removal device (CRD 200; 2 mm) was installed between the suppressor eluent out and the conductivity detector eluent in ports. Ammonium, calcium, magnesium, potassium, and sodium were resolved using a CS-12A separator column (2 by 250 mm) protected by a CG-12A guard column (2 by 50 mm). Isocratic methylsulfonate at 20 mM was produced using a methylsulfonic acid-based eluent-generated cartridge and pumped at 0.25 ml/min. Suppressed conductivity detection using a Dionex CERS-500 2-mm suppressor operated in eluent recycle mode with an applied current of 15 mA. Ammonium standards ranging from 1 μM to 1 mM (1 μM, 10 μM, 50 μM, 100 μM, 500 μM, and 1 mM) were run along with samples. A standard curve was generated by fitting a quadratic curve to standard measurements.

### C/N measurements.

For defined medium conditions, 16 5-ml cultures (either in pyruvate defined medium with or without NH_4_Cl or glycerol defined medium) were grown to an OD_600_ between 0.5 and 0.7. The 16 cultures were divided into two sets of eight cultures. All eight cultures in a set were poured into a single 50-ml conical tube. Samples were then centrifuged at 6,000 × *g* for 10 min at 4°C. Pellets were then washed twice with 25 ml PBS, with centrifugation in between. After the second wash, each pellet was resuspended in 1.2 ml PBS, which was divided among two 1.5-ml centrifuge tubes in 600-μl aliquots (for a total of four samples/condition). After centrifugation at 16,000 × *g* for 1 min, supernatants were pipetted off and pellets were flash frozen in liquid nitrogen and stored at −80°C. Frozen samples were lyophilized, and ∼50 μg (for carbon measurement) and ∼700 μg (for nitrogen measurement) of each sample were weighed into an organic elemental analysis (OEA) lab tin capsule (pressed, ultraclean, C61480.096P). Carbon and nitrogen were measured separately due to differing sensitivities of the instrument. Each sample was combusted in a Thermo Fisher EA IsoLink combustion system by oxidation at 1,020°C over tungstic oxide, followed by reduction over elemental copper packed in the same furnace. The generated CO_2_ and N_2_ carried by a continuous helium flow (100 ml/min) were subsequently passed through a water trap and then a 5-Å molecular sieve gas chromatograph (GC) at 50°C. The GC was used to separate N_2_ from CO_2_. Carbon and nitrogen were then diluted with helium in a Conflo IV interface/open split prior to entering the Thermo Fisher Delta V isotope ratio mass spectrometry (IRMS) system for analysis. Depending on the configurations of the IRMS, either CO_2_ or N_2_ was measured for its total abundance. Integrated peak areas for both CO_2_ and N_2_ were calibrated by running urea standards, and empty tins were included as blanks. A Student *t* test was used to generate *P* values comparing conditions.
