# Supplementary material for: Aggregation of Nontuberculous Mycobacteria Is Regulated by Carbon-Nitrogen Balance
Source: mBio. 2019 Aug 13;10(4):e01715-19. doi: 10.1128/mBio.01715-19 (PMC6692514; doi:10.1128/mBio.01715-19)
Supplement: TABLE S1 [file mBio.01715-19-st001.docx]

| **Table S1**. Strains, plasmids, and primers used in this study. | | |  |  |  |
| --- | --- | --- | --- | --- | --- |
|  |  | |  |  |  |
| Strains | Notes | | Reference |  |  |
| *Mycobacterium smegmatis* MC^2^155 | WT strain | | (1) |  |  |
| *M. smegmatis*/pJV53 | Used for recombineering | | This study |  |  |
| *M. smegmatis*::pMH94 EV | Empty pMH94 vector integrated into WT *M. smegmatis* | | This study |  |  |
| *M. smegmatis* Passage 40 isolate | From serial passage experiment | | This study |  |  |
| *M. smegmatis* Passage 60 isolate | From serial passage experiment | | This study |  |  |
| *M. smegmatis* Pass60::pMH94 EV | pMH94 vector integrated into *M. smegmatis* Passage 60 isolate | | This study |  |  |
| *M. smegmatis* Pass60::pMH94-opp | pMH94-opp vector integrated into *M. smegmatis* Passage 60 isolate | | This study |  |  |
| *M. smegmatis* Δ*opp* | MSMEG_0639-MSMEG_0643 deleted via recombineering, pJV53 cured | | This study |  |  |
| *M. smegmatis* Δ*opp*::pMH94 EV | pMH94 vector integrated into *M. smegmatis* Δ*opp* | | This study |  |  |
| *M. smegmatis* Δ*opp*::pMH94-opp | pMH94-opp vector integrated into *M. smegmatis* Δ*opp* | | This study |  |  |
| *M. smegmatis* Δ*kdpD* | MSMEG_5395 deleted via recombineering, pJV53 cured | | This study |  |  |
| *M. smegmatis* Δ*MSMEG_6497* | MSMEG_6497 deleted via recombineering, pJV53 cured | | This study |  |  |
| *Mycobacterium fortuitum* ATCC 6841 |  | | ATCC strain |  |  |
| *Mycobacteria abscessus* ATCC 19977 |  | | ATCC strain |  |  |
| NTM0253a | *M. abscessus* subp. *abscessus* smooth colony clinical isolate from Cystic Fibrosis patient | | L. Caverly |  |  |
| NTM0711a | *M. abscessus* subp. *abscessus* smooth colony clinical isolate from Cystic Fibrosis patient | | L. Caverly |  |  |
| NTM0253b | *M. abscessus* subp. *abscessus* rough colony clinical isolate from Cystic Fibrosis patient | | L. Caverly |  |  |
| NTM0711b | *M. abscessus* subp. *abscessus* rough colony clinical isolate from Cystic Fibrosis patient | | L. Caverly |  |  |
|  |  | |  |  |  |
| Plasmids | Notes | | Reference |  |  |
| pMQ30 | Plasmid containing gentamicin resistance cassette | | (2) |  |  |
| pJV53 | Recombineering vector | | (3) |  |  |
| pMH94 | Integration vector | (4) | | | |
| pMH94-*opp* | Integration vector in which the 5-gene opp operon (MSMEG_0643-MSMEG_0639) plus 207 bps upstream of MSMEG_0643 was cloned into the xbaI site. | This study | | | |
| Primers | Sequence | | Notes | |  |
| Msmeg opp 5’ F | ATCGGTCCGCTCGACATC | | Used to amplify 5’ and 3’ flanking regions of the opp operon for opp deletion fragment | |  |
| Msmeg opp 5’ R xbaI* | GATCTCTAGATGATTCCCCTTGATTTCCAA | |  |  |  |
| Msmeg opp 3’ F NotI* | GATCGCGGCCGCTCAGGGGTAAGGCTGGAACG | |  |  |  |
| Msmeg opp 3’ R | GAACCCAAACCCCTGACAC | |  |  |  |
| opp gibson gent F | GCATCCAAGTTTGGAAATCAAGGGGAATCATCTAGAGATCCTAAATACATTCAAATATGT | | Used to amplify gentR cassette for opp deletion fragment | |  |
| opp gibson gent R | GCAGAACCCGTTCCAGCCTTACCCCTGAGCGGCCGCGATCTTAGGTGGCGGTACTTGGGT | |  |  |  |
| Msmeg6497 5’ F | cgtcgacgtagacggtcttc | | Used to amplify 5’ and 3’ flanking regions of MSMEG_6497 for 6497 deletion fragment | |  |
| Msmeg6497 5’ R xbaI* | GATCTCTAGAACAAATCCTGTCAGCTGTCA | |  |  |  |
| Msmeg6497 3’ F NotI* | GATCGCGGCCGCGCGTGCGTGAAATGCCGAGC | |  |  |  |
| Msmeg6497 3’ R | GCTGATGGACGAACCGTTC | |  |  |  |
| 6497 gent F | GCGATTTAATTTCAGGTGGCAGTAATTGATTGACAGCTGACAGGATTTGTCTAAATACATTCAAATATGT | | Used to amplify gentR cassette for 6497 deletion fragment | |  |
| 6497 gent R | TCGGCGTGCGGTCCGCCGGATGCGGTGTCGGCTCGGCATTTCACGCACGCTTAGGTGGCGGTACTTGGGT | |  |  |  |
| Msmeg5395 5’ F | AATCTGGGACCGGAAAGC | | Used to amplify 5’ and 3’ flanking regions of MSMEG_5395 for 5395 deletion fragment | |  |
| Msmeg5395 5’ R gent gib | TTATTGTCTCATGAGCGGATACATATTTGAATGTATTTAGTGTCGAGAGCGATGTTGAGT | |  |  |  |
| Msmeg5395 3’ F gent gib | AGTGATGCACTTTGATATCGACCCAAGTACCGCCACCTAAAACCTGTCGGTACGAGGCTA | |  |  |  |
| Msmeg5395 3’ R | CGCGTAAATAGTGGGTCTCG | |  |  |  |
| 5395 gent F | CTAAATACATTCAAATATGT | | Used to amplify gentR cassette for 5395 deletion fragment | |  |
| 5395 gent R | TTAGGTGGCGGTACTTGGGT | |  |  |  |
| opp pMH94 comp xbaI F | GATCTCTAGACCGTCACACTCTCGTCATAGTC | | Used to amplify *opp* operon from WT *M. smegmatis* and clone into pMH94 | |  |
| opp pMH94 comp xbaI R | GATCTCTAGACAGTACCCGCCGCAATATC | |  |  |  |
|  |  | |  | |  |
|  |  | |  |  |  |
|  |  | |  | |  |

*Although these primers include restriction sites, Gibson assembly was used to create the respective linear fragments for recombineering

References

1. **Snapper SB, Melton RE, Mustafa S, Kieser T, Jacobs WR**. 1990. Isolation and characterization of efficient plasmid transformation mutants of *Mycobacterium smegmatis*. Mol. Microbiol. **4**:1911–1919.

2. **Shanks RMQ, Caiazza NC, Hinsa SM, Toutain CM, O’Toole GA**. 2006. *Saccharomyces cerevisiae*-based molecular tool kit for manipulation of genes from gram-negative bacteria. Appl. Environ. Microbiol. **72**:5027–5036.

3. **Van Kessel JC, Marinelli LJ, Hatfull GF**. 2008. Recombineering mycobacteria and their phages. Nat. Rev. Micro. **6**:851–857.

4. **Lee MH, Pascopella L, Jacobs WR, Hatfull GF**. 1991. Site-specific integration of mycobacteriophage L5: integration-proficient vectors for *Mycobacterium smegmatis*, *Mycobacterium tuberculosis*, and bacille Calmette-Guérin. Proc. Natl. Acad. Sci. USA **88**:3111–3115.
